# 

**Published:** 1875-07

**Authors:** 


### CONTENTS.

PAGE.

**CONTRIBUTIONS.**

Recreation without Damnation. Ben. H. Pratt... 29
Man's Place in Creation. P. H. Hayes, M. D........ 31
Habit. B. Browne ........ 33
California. Dr. Gleason........ 34

**MEDICAL ITEMS AND NEWS.**

Local Anæsthesia—Sulphate of Cadmium in Gonorrhea—Wine of Beef and Iron—Iodine Caustic—Ozœna—Anti-Constipation Pills—"Dead Shot Pills"—Snow as a Hæmostatic—Hemorrhoids—To prevent Surgical Fever—Nelaton's Method of Resuscitation from Chloroform—Local Anodyne—Hypodermic Injection of Ergot—Sulphides in Boils--Cold Powder--Liquid Dentifrice--Lotion for Prurigo—Rheumatism—Croton Oil Paint—Cheap Fumigator—Carbolic Acid Inhalations—Cauterization in Hydrocele—Anæsthesia by Injection of Chloral—Sciatica—An Improved Poultice—Calamus in Dysentery—Cyanides in Rheumatism—Cod-Liver Oil Mixture—Hemorrhage in Extracting Teeth—Epilepsy—Hypodermic Injection of Ergotine in Varicocele—Antagonism between Choral Hydrate and Calabar—Ringworm—Burns and Scalds—Chocolate in Intestinal Catarrh—Catarrh—Guarana in Headache—Rattle Snake Bite—Whooping Cough—Diphtheria........36 to 43

**OUR CORRESPONDENCE.**

Eleven Letters with Answers ........ 44

PAGE.

**HOUSEHOLD HINTS AND RECIPES.**

Paste that Adheres to Tin—Bleaching Sponges—Imitate Ground Glass—Starch Enamel—Warts—Cement for Brass and Wood—Experiment—Linens, to Wash—Cologne Water—Sweating Feet—Cabbage and Ham Salad—Gutta Percha Cement—Quality of Pears—To Discover Colored Wines—Mathematical Fact—Match under Microscope—Detection of Fats in Butter—Nickel Plating—India Rubber Cement—Paste—Colorless Varnish—Basket Plants—To Keep Eggs—Wholesome Meat........46 to 48

**EDITORIAL.**

Angling........ 49

Accidents to the eye........ 52

The Laryngoscope........ 53

**EDITORIAL CHIT-CHAT.**

Holloway's Asylum—Vehicle for Medicine—Vacation—After a Ball—Fifth Ward Steamer—Mean Patient—Double Headed Trout—Father Stack—The Fourth—Dr. Gleason's Return—Strong Inquiry—Flood the Low Ground—Exercise, the Flower Garden........55 to 56

